# Triqler for Protein Summarization of Data from Data-Independent Acquisition Mass Spectrometry

**DOI:** 10.1021/acs.jproteome.2c00607

**Published:** 2023-03-29

**Authors:** Patrick Truong, Matthew The, Lukas Käll

**Affiliations:** † Science for Life Laboratory, School of Engineering Sciences in Chemistry, Biotechnology and Health, Royal Institute of Technology − KTH, Solna 17121, Sweden; ‡ Chair of Proteomics and Bioanalytics, Technical University of Munich (TUM), Freising 85354, Germany

**Keywords:** mass spectrometry, protein summarization, Bayesian hierarchical modelling, label-free quantification, data-independent acquisition mass spectrometry,, benchmark, mathematical methods

## Abstract

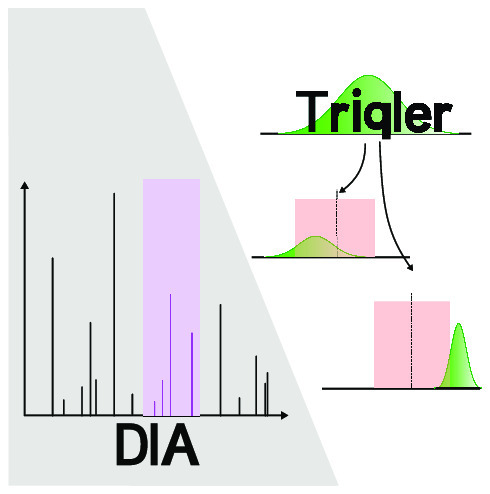

A frequent goal, or subgoal, when processing data from a quantitative shotgun proteomics experiment is a list of proteins that are differentially abundant under the examined experimental conditions. Unfortunately, obtaining such a list is a challenging process, as the mass spectrometer analyzes the proteolytic peptides of a protein rather than the proteins themselves. We have previously designed a Bayesian hierarchical probabilistic model, Triqler, for combining peptide identification and quantification errors into probabilities of proteins being differentially abundant. However, the model was developed for data from data-dependent acquisition. Here, we show that Triqler is also compatible with data-independent acquisition data after applying minor alterations for the missing value distribution. Furthermore, we find that it has better performance than a set of compared state-of-the-art protein summarization tools when evaluated on data-independent acquisition data.

## Introduction

Mass spectrometry (MS)-based proteomics enables efficient detection of proteins in complex mixtures. There are several different techniques to make the technology quantitative.^1,2^ Out of these, label-free quantification (LFQ)^3^ has the advantage that it can smoothly handle large sample sizes. For data from LFQ experiments, just as in other quantification schemes, there exists a plethora of processing options, all containing several processing steps, each subject to their different error sources, all affecting the result of the processing.

We have previously designed a hierarchical Bayesian model, Triqler, able to control for errors from both the identification and the quantification process in LFQ experiments.^4^ By integrating the error probabilities from identification and quantification, one can obtain better accuracy in calling differentially abundant proteins. Triqler does so by assuming that peptide abundances follow a probability distribution. This abundance distribution is initiated according to a prior distribution, but updated on the basis of each sample’s registered peptide abundance values. This results in peptide abundance distributions that are integrated into protein abundance and subsequently fold change distributions. In the processing, Triqler weights in information on differences in peptide abundances within sample groups and search engine identification error probabilities. Data that indicate uncertainty result in wider abundance distributions, while certainty results in tighter abundance distributions. Triqler also integrates the resulting fold change distributions to derive posterior probabilities for each protein having an actual fold change larger than a preset threshold value. These posterior probabilities are also averaged into *q* values.^4^

Triqler was originally designed for handling LFQ data from data-dependent acquisition (DDA). However, in principle, once peptide abundances and identities are estimated, there are only small differences between data from DDA and data-independent acquisition (DIA) mass spectrometry.^5^ Here, we set out to investigate Triqler’s ability to summarize protein concentrations from peptide abundances derived from DIA data. We used the LFQBench from Navarro et al.^6^ for the evaluation. In the original benchmark, Navarro et al. included a comparison of different protein summarization strategies and found that the so-called Top3 method generally resulted in lower variance and better quantification accuracy than did the built-in methods from OpenSwath, SWATH2.0, Skyline, Spectronaut, and DIA-Umpire.^6^ However, there are reasons to believe that more sophisticated methods would yield better protein quantification than the Top3 method. Simple summarization methods based on mean and median peptide intensity have been shown to produce unreliable protein abundance estimates,^7^ and more advanced summarization strategies for LFQ data have been proposed in the literature.^8,9^ Summarization techniques such as PQPQ,^10^ MSstats,^11^ Diffacto,^12^ MSqRob2,^13^ and Triqler^4^ have all been shown to outperform Top3, and there are no theoretical reasons why the stated methods would not perform well for DIA data. Hence, we found it apt to modify Triqler to handle DIA data and to benchmark it against a set of state-of-the-art protein summarization methods.

## Materials and Methods

### LFQBench Mass Spectrometry Data

We downloaded the LFQBench data set^6^ from ProteomeXchange (PXD002952). Here, we used the TripleTOF6600 section of the study, which was harvested with a setup of 32 fixed windows MS2-windows. We also restricted ourselves to the low ratio difference samples, referred to as the HYE124 hybrid proteome samples in the original study. These consist of triplicates of sample A, composed of tryptically digested proteins from 65% w/w HeLa, 30% w/w yeast, and 5% w/w *E. coli* cells, and triplicates of sample B, composed of 65% w/w, 15% w/w yeast, and 20% w/w *E. coli* proteins. Samples from HYE110 and the TripleTOF5600 section of PXD002952 were omitted in this study. Further details about mass spectrometric instrumentation and data acquisition are available in Navarro et al.^6^ The .wiff files were converted to .mzML files in a centroided format using msconvert (using Windows OS msconvert version 3.0) with peakPicking filter msLevel = 1-.

### LFQBench Sequence Database

Uniprot FASTA files with one protein sequence per gene were downloaded for each species (UP000005640, UP000000625, and UP000002311, acquired on 2021-06-16). The unfiltered FASTA files contained 20 590 human proteins, 6046 yeast proteins, and 4373 *E. coli* proteins. To reduce the effect of the different protein inference strategies for the tested protein summarization tools, a modified FASTA file, without shared peptides, was used for the database search. The filter removed protein sequences with shared peptides so that the final database did not contain any two proteins sharing tryptic peptides longer than seven amino acids. After filtering, the FASTA file contained 20 302 proteins (288 human proteins fewer proteins than in an unfiltered database), 5848 yeast proteins (198 yeast proteins fewer proteins than in an unfiltered database), and 4306 *E. coli* proteins (67 *E. coli* proteins fewer proteins than in an unfiltered database). Replacing the I/L amino acids to handle the mass equivalence did not result in any considerable differences (see Table S1). We also added pseudoreverse sequences to the database as decoys for target-decoy analysis using OpenSwathDecoyGenerator.

### Cancer Proteomics Data

We also downloaded two DIA data cancer proteomics-related data sets from ProteomeXchange, with accession numbers PXD004684 and PXD022992. The first set consists of data from four frozen lung squamous cell carcinomas and four adjacent tissues harvested with a Thermo QExactive Plus.^14^ The second set consists of data from a comparison of three primary cell lines (SK-MEL-1, A375, and G-361) to three metastatic (RPMI-7951, SH-4, and SK-MEL-3) cell lines analyzed (two samples per cell line) by a Thermo Orbitrap Fusion Lumos Tribrid.^15^

### General Workflow

We used three separate strategies to generate peptide abundances from the DIA runs. First, we used a spectral library consisting of selected spectra from separate DDA runs, and we refer to this workflow as ID; second, we searched pseudospectra generated directly from the DIA data, and we will refer to this workflow as PS; and third we applied a DIA-NN centered pipeline that also operated directly on DIA data. We will describe the parameter choices of these methods below.

### DDA Identification-Driven (ID) Spectral Library

For the ID workflow, we searched the LFQBench-provided DDA runs with MSFragger^16^ with default settings, and a spectral library was constructed with EasyPQP^17^ with the default setting. No transition refinement was used. OpenSwatchDecoyGenerator was used to generate decoys for the spectral library with a pseudoreverse method. The DIA data were searched with the spectral library through OpenSwath Workflow, and the “m_score” was computed using PyProphet.^18^ The “m_score” cutoff for a user-specified peptide identification false discovery rate was computed with SWATH2STATS.^19^ This process resulted in a set of detected peptides together with their assessed peptide identification accuracies and abundance estimates (Table S2 shows the number of identified peptides and proteins).

### Pseudospectra Generation (PS) Spectral Library

For the PS workflow, we also used the FragPipe software, employing DIA-Umpire to extract pseudospectra from the DIA data. The DIA-Umpire parameters were set to default values. The pseudospectra were subsequently searched using MSFragger with default settings. A spectral library was built from the resulting PSMs using easyPQP with default settings. DIA-NN^20^ (version 1.7.12) was used for peptide quantifications with default settings.

### Directly Extracted Peptide Abundances

For this workflow, we used the quantms nf-core pipeline (https://nf-co.re/quantms), which converts raw files to mzML with ThermoRawFileParser^21^ and subsequently extracts peptide abundances using DIA-NN.^21^ The pipeline generated decoys for FDR calculations, which were discarded after DIA-NN processing. To circumvent the lack of decoys in output for Triqler, we concatenated shuffled entrapment sequences in the FASTA database.^22^ This workflow was used with the two cancer proteomics-related data sets.

### Protein Summarization

The peptide quantities were summarized to proteins using the average of the three most intense peptides (we call it Top3), MSstats, MSqRob2, and Triqler. This is done for all three pipelines.

### Top3

We implemented a short script that takes the average of the three most abundant PSMs for each protein and sample. In samples only having two PSMs, these were also included, still represented by their average. Proteins with one or zero PSMs per sample were excluded. PSMs were filtered at a 1% PSM-level FDR before the Top3 protein summarization was performed.

### MSstats

We installed MSstats version 3.18.5 using R/Bioconductor (available at https://www.bioconductor.org/packages/release/bioc/html/MSstats.html). MSstats uses feature-level data, allowing for multiple PSM hits per peptide identification. We use the disaggregate function to disaggregate the PSMs to fragment-level data. We filtered so that every protein had at least 2 peptides and a maximum of 10 peptides, and we thresholded with an “m_score” that corresponds to a peptide identification FDR lower than 1% for both ID and PS pipelines. For the cancer data set, we allowed a maximum of 100 peptides. In the PS pipeline and nf-core pipeline, the data were filtered on the “Q.Value” columns from the DIA-NN output file. In the ID pipeline, we computed an “m_score” corresponding to a 1% FDR and used this “m_score” to filter the data. MSstats was run using the MSstats command “dataProcess”. The significance testing between conditions was performed using the MSstats function “groupComparison”.

### MSqRob2

We installed MSqRob2 version 0.9 using R/Bioconductor (available at https://github.com/statOmics/msqrob2). MSqRob2 takes peptide-level input. The output from OpenSwath and DIA-NN is at the PSM-level. We select the top PSM hit as our peptide and filter the data on 1% peptide level FDR to get peptide-level data. The highest-scoring PSMs are selected by the highest “m_score” for OpenSwath and highest “CScore” for DIA-NN. MsqRob2 was run using the MSqRob2 command “msqrob”, where the contrast was set to “condition”. MSqRob2 uses “lme4” to construct a linear-mixed model with random effect, but without fixed effect.

### Limma Analysis on DIA-NN Protein Groups

We downloaded limma^23^ version 3.50.3 from Bioconductor. We applied limma with default parameters to the protein groups inferred from DIA-NN in the PS pipeline.

### Triqler

We downloaded Triqler from https://github.com/statisticalbiotechnology/triqler. We used Triqler v0.6.1 for the tests described in this work. We selected a lower bound estimate for the “fold_change_eval” parameter as described in The and Käll,^24^ which ended up as 0.76 for the spectral library data and 0.51 for the pseudospectra enabled data.

As Triqler’s model accounts for the assessed uncertainty in the prior identification steps of the data, it does not threshold the PSMs but instead takes all of the PSMs as input. The “searchScore” column should reflect increasing certainty in PSMs. Therefore, we apply the negative log-transform to the “m_score” or “Q.Value” to indicate “searchScore” for the two different pipelines.

When evaluating Triqler, we estimated a fold-change valuation thresholds using a previously described heuristic that ensures that 99% of the probability distribution of the normal distribution is contained,^24^ as
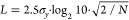
1where σ_*y*_ is the standard deviation of the protein prior in log_10_ abundance units, and *N* is the number of samples in the group. Triqler estimates and prints this minimum advisible fold change threshold by default, and it can be retrieved from Triqlers standard output. Also, Triqler removes any peptides that are attributed to more than one protein.

### Multiple Hypothesis Correction

There was a slight difference in some of the benchmarking metrics, as the multiple test correction is performed with *q* value for Triqler and Top3, while MSstats and MSqRob2 use Benjamini–Hochberger^25^ corrections. The *q* value approach aims to give an unbiased estimator of FDR, while the Benjamini–Hochberger approach estimates the upper bound of the FDR (and will therefore result in an FDR equal to or higher than the *q* value). As a consequence, the statistics from MSstats and MSqRob2 should be more conservative than the estimates from Triqler and Top3.^26^

## Results

To establish that Triqler is useful when evaluating DIA data, we first examined the DIA data to establish that the abundance values derived from such processes are in line with the assumptions that Triqler makes about input data. Second, we benchmarked Triqler against a set of state-of-the-art methods for protein summarization. For both tasks, we used selected parts of the LFQBench data set, which we processed in two different ways. We used a pipeline with a DDA identification-driven (ID) spectral library, as well as one using a Pseudospectra generation (PS) spectral library (see Materials and Methods).

### Validations of Properties of DIA Peptide Abundance

DIA data are assumed to encompass a larger dynamic range than DDA data. This could affect one of Triqler’s assumptions, that the noise structure is mainly multiplicative, that is, that the standard deviation within a sample group is proportional to its mean. When investigating all of the peptide abundance measurements at a 1% PSM-level identification false discovery rate (FDR) from the TripleTOF6600 section of the LFQBench data set, we found a relatively linear relationship between the standard deviation and the mean (Figure S1A–D).

However, when estimating the distribution of missing values with Triqler, we noted that the lower number of missing values in DIA as compared to DDA (<2% for DIA) led to parameter estimates out of the expected range. We hence introduced an alternative estimation procedure for DIA data. Instead of fitting a censored normal distribution function over all XIC values as for DDA data, we use the mean value of the missing values to fit a censored normal distribution function (see Figure S2). The alternative curve-fitting procedure is enabled by a command line option in Triqler named “--missingValuePrior DIA”.

### Harmonization of Protein Inference Procedures

Encouraged by the finding that peptide abundances from DIA data have properties similar to those of DDA data, we wanted to see how well Triqler works in practice. Particularly, we wanted to compare the performance of Triqler to that of other protein summarization methods. However, to do so we first needed to establish some principles for how to benchmark summarization methods on data from whole-cell mixtures, when comparing methods that use different protein inference procedures.^27^ When reporting the number of differentially abundant proteins in data sets from mixtures from whole-cell extracts, a protein inference scheme that infers any protein containing a detected peptide will report more differentially abundant proteins than a more restrictive scheme that just reports a parsimonious set of proteins. There are no restrictive mechanisms detecting situations where nonpresent proteoforms are reported as long as they are reported with protein abundance rates compatible with the right proteome. To alleviate, or at least minimize, this problem from our comparison, we restricted the searched sequence database by removing proteins with shared peptides in such a manner that no two proteins shared a tryptic peptide longer than 7 amino acids. This operation aims at a fairer comparison of protein summarization regardless of the protein inference method.

### Distributions of the Estimated Fold Changes

**Figure 1:**
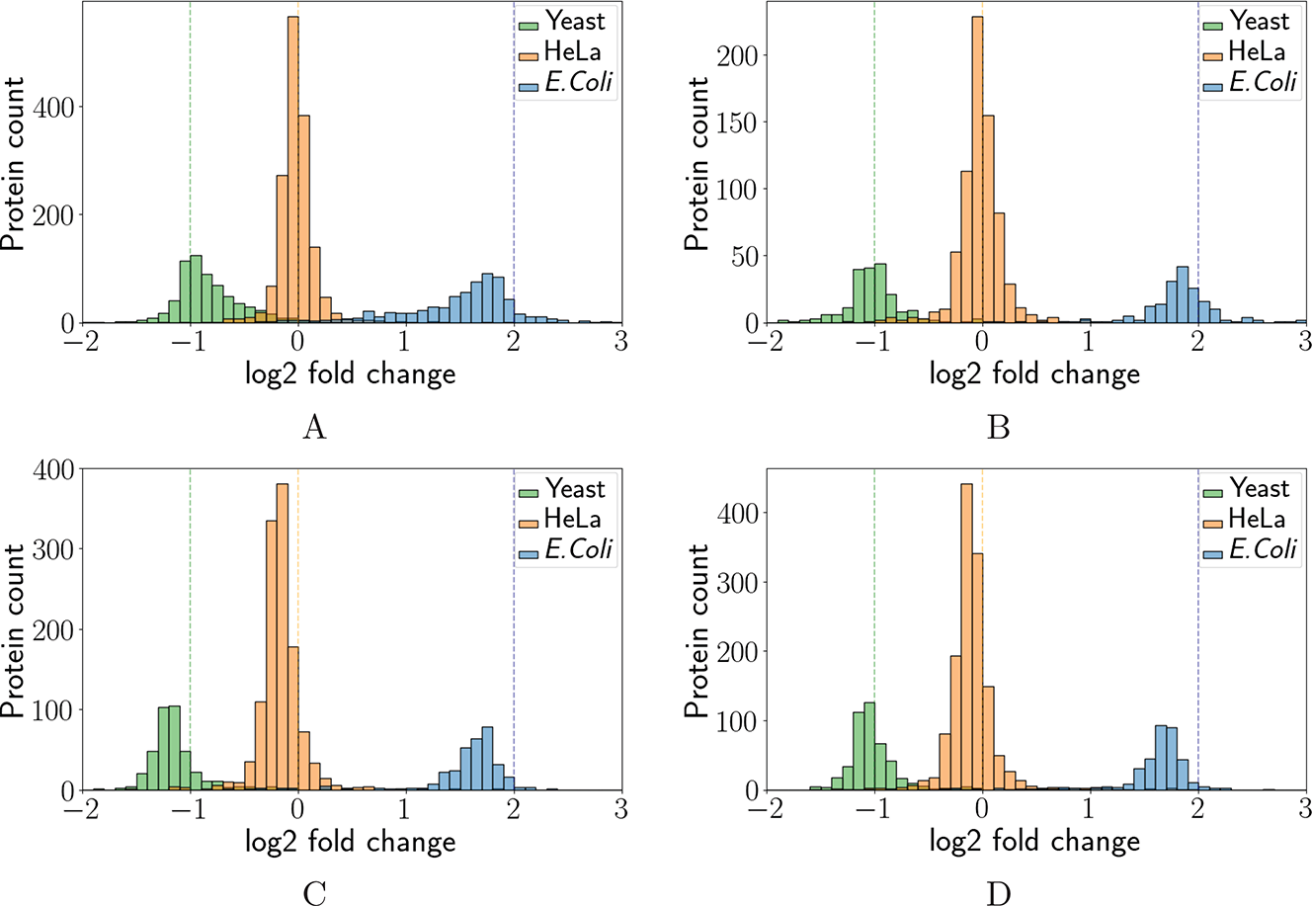
Comparison of reported fold change distributions. The summarized protein abundances from the ID spectral library pipeline for (A) Triqler, (B) Top3, (C) MSstats, and (D) MSqRob2 were binned and plotted as histograms. The dashed lines indicate the pipetted log2-fold change ratio between a specie and HeLa samples. We noted that the empirical densities of the protein counts are less biased for Triqler and Top3 than for MSstats and MSqRob2, as the apex of their distribution was found closer to the true fold change difference (see Figure S7 for the reported fold change distributions for both ID and PS workflows). For these histograms, we included all of the summarized proteins and did not remove any proteins based on the significance or fold change thresholds.

We applied Triqler, MSstats, MSqRob2, and Top3 (see Materials and Methods) to the peptide abundances derived from the ID and PS workflows from the varying concentrations of *E. coli* and yeast concentrations in a background of HeLa-cells of the LFQBench set. Although Triqler is not meant as a tool to give point estimates, we here used Triqler’s maximum a posteriori probability (MAPs) as point estimates of protein abundance to enable comparisons to other methods. Also, for the comparisons, we removed the methods’ fold change selection mechanisms. For an overview of the results, we made histograms of the methods’ estimated protein-level fold changes (Figures 1 and S7).

**Figure 2:**
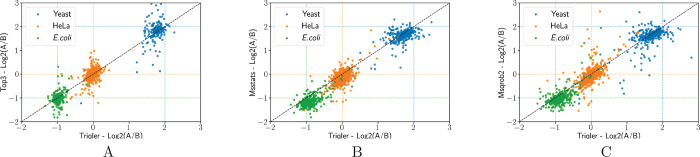
Comparison of the summarized protein fold changes between the methods. The comparison between the distributions of the MAP estimates from Triqler and (A) Top3, (B) MSstats, and (C) MSqRob2 for the data in the ID pipeline. The actual pipetted fold changes are indicated by dashed lines. Proteins not quantified by both compared methods were excluded from the plots. See Figure S3 for protein-level results for both ID and PS workflows.

Triqler and Top3 appear to have less fold change bias than MSstats and MSqRob2; that is, the apex of the distributions is centered more closely to the lysate mixture rates. MSstats and MSqRob2 have distribution apexes that, for each species, are centered at lower fold changes than the true lysate mixture rates. Reprocessing the data with replaced labels led to the reversed result, that is, the apexes centered around values higher than the pipetted mixture rates for both MSstats and MSqRob2 (data not shown). We also observe that the apex of the distributions has higher values for Triqler than for the other methods in Figure 1. We provide comparisons of the fold change estimates in Figures 2 and S3.

As visible in Figure 1, Triqler’s fold change distributions have longer tails for the estimated yeast and *E. coli* than the compared methods. These values are explained by proteins quantified with less certainty. Proteins with correctly estimated fold changes are identified by more peptides than those differing significantly from their expected fold change (data not shown). The technical aspect of this is explained by how Triqler computes the posteriors for differentially abundant proteins; with better data, more confidence is put on data and less confidence is put on the prior distributions, resulting in narrower fold change distributions with means more centered at the observed mean values.

### Comparison of Ability to Discriminate Differentially from Equivalently Abundant Proteins

**Figure 3:**
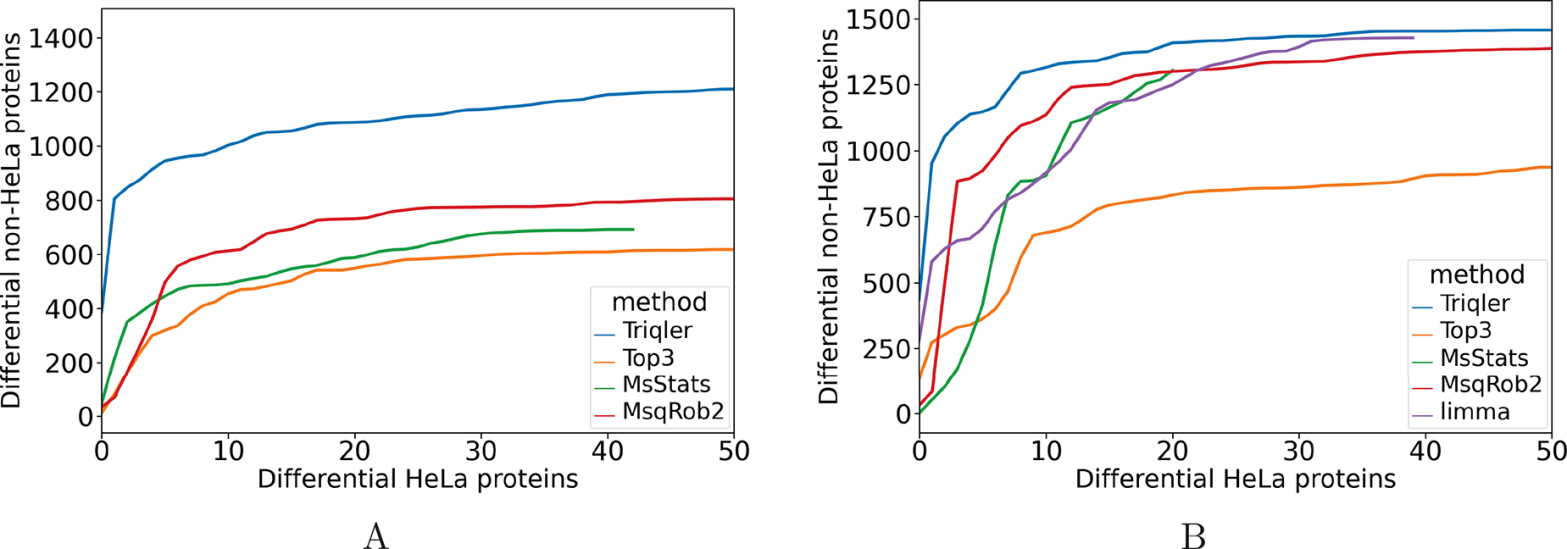
Comparison of the ability to differentiate proteins with differential abundance between conditions. We plotted the number of reported differentially abundant *E. coli* and yeast proteins as a function of the number of proteins from the HeLa background when sorting according to significance for the (A) ID pipeline and (B) PS pipeline. For the test, we selected a fold change evaluation of 0.51 for Triqler and a fold change threshold of 0.51 for Top3, MSstats, and MSqRob2. See Figures S4–S6 for the number of differentially abundant proteins for each proteome.

As the first quantitative test of performance, we compared the methods’ reported number of differentially abundant *E. coli* and yeast proteins as a function of the number of HeLa proteins (Figure 3). As the former two lysates were injected in different concentrations and the HeLa was at constant concentration over the sample groups, a higher number of non-HeLa proteins for a similar number of HeLa-proteins is seen as better performance. Overall, Triqler reports more such expected differentially abundant non-HeLa proteins per HeLa protein than the compared methods for both the peptide abundances generated by the ID spectral library and the PS spectral library pipelines. The results can be found in Figure S4. We also ran a second experiment where we removed shared peptides after matching, to ensure that the order of removal of the shared peptides does not affect performance. However, we found no large differences between removing peptides before or after database searching (Figure S6).

### Comparison of Statistical Calibration

**Figure 4:**
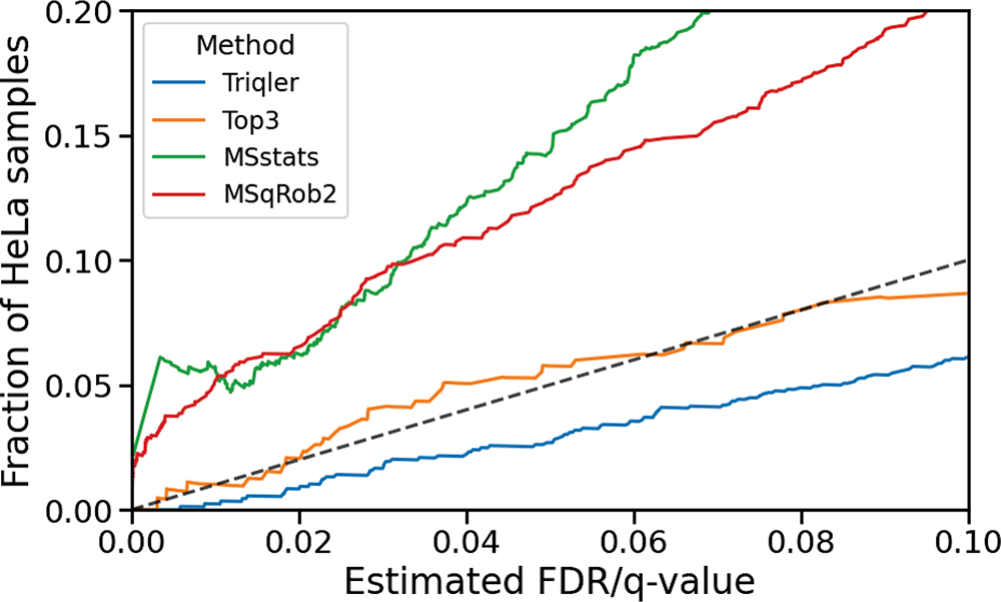
Comparison of calibration of the compared summarization methods. We plotted the fraction of reported differentially abundant HeLa proteins as a function of the *q* value threshold for protein abundances without any restrictions on the fold change. We used the *q* value for Triqler and Top3 and the Benjamini–Hochberg corrections for MSstats and MSqRob2.

Further, we tested the statistical calibration of the summarization methods. We hence investigated the relationship between the fraction of wrongly reported differentially abundant proteins (i.e., the fraction of HeLa proteins among all reported differentially abundant proteins) and each inference method’s estimated false discovery rate (see Figures 4 and S8). We observed that Triqler was better calibrated (closer to the diagonal line) than were MSstats and MSqRob2. Top3 showed an even better calibration than Triqler but, as previously demonstrated, has a much lower sensitivity.

### Benchmark on Cancer Proteomics-Related Data

**Figure 5:**
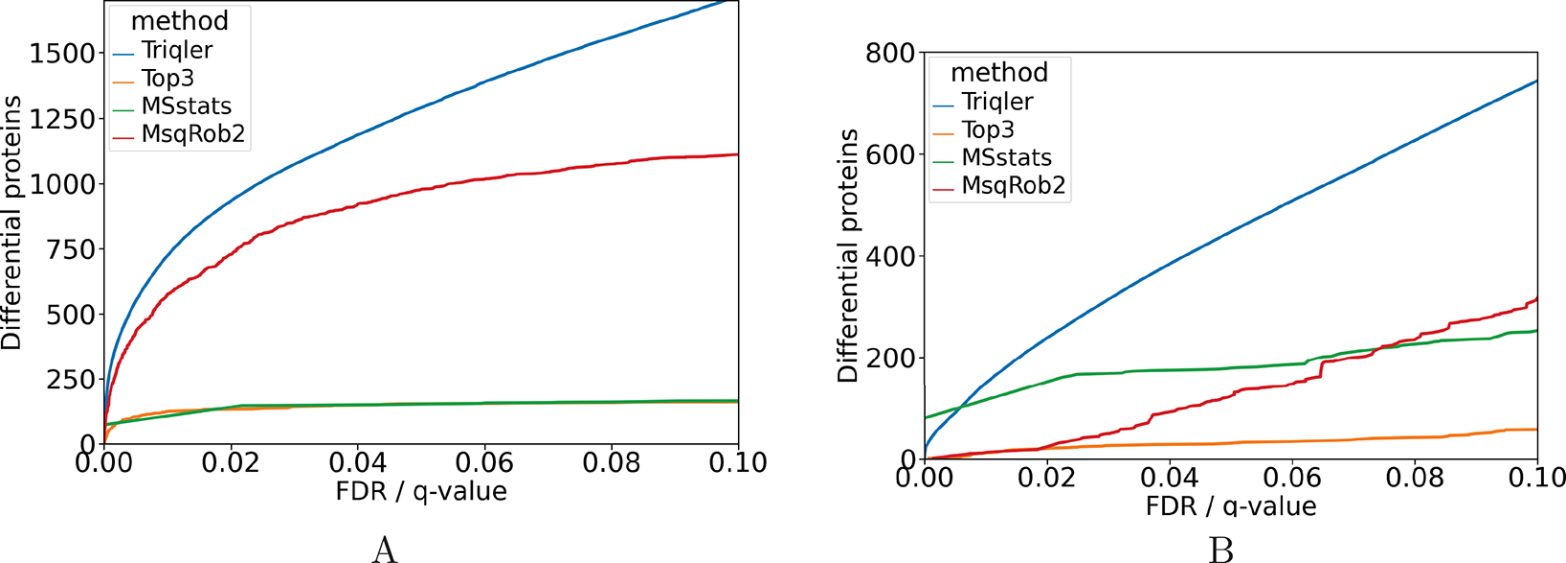
Reported performance of the compared methods on two cancer proteomics-related data sets. We plotted the reported differentially abundant proteins as a function of their reported FDR/*q* value for the (A) Stewart et al. set (lung squamous cell carcinomas vs adjacent tissues) and (B) Gao et al. set (primary vs metastatic cell lines).

To further compare the different protein inference methods, they were finally tested on two sets of less engineered data, sets without known relative abundance differences. Data were downloaded from Stewart et al., a set consisting of DIA data contrasting lung squamous cell carcinomas to adjacent tissues,^14^ and from Gao et al., a set comparing primary to metastatic cell lines.^15^ Peptide abundances were derived with the nf-core quantms pipeline followed by our protein summarization strategies and plotted the number of differentially abundant proteins as a function of each method’s estimated quantitative protein-level FDR or *q* value. We used a fold change evaluation of 0.92, respectively, 0.63 for Triqler, based on its lower bound estimate. We used the same lower bound estimate as a fold change threshold when reporting the performance of the compared methods. As can be seen in Figure 5, Triqler reports more differentially abundant proteins than the other methods for any estimated *q* value threshold.

## Discussion

Here, we have shown that Triqler operates well for DIA data, despite being originally intended for DDA data. We also find that Triqler outperforms other protein summarization methods on an engineered benchmark set, in terms of both sensitivity and accuracy in its error estimates. Triqler was also able to detect a higher number of differentially abundant proteins at a more accurately reported false discovery rate than the compared methods. The absence of filtering and imputation steps before Triqler benefits the analysis by making it both more user-friendly by reducing parameter choices and inducing less bias into the result.

The analytes in shotgun proteomics are peptides and not proteins or proteoforms. Nevertheless, most users of mass spectrometry use and will continue to find reasons to report findings on a protein level. It makes sense to put efforts into a better understanding of which protein inference tools to use at what occasion and how to summarize peptide abundances into protein relative concentration values. Also, protein summarization gives lower variance than peptide-level analysis, and it reduces the number of hypotheses tested and reduced the number of missing values, which can have a major impact on the quality of the analysis.^28^

One important remark is that the sequence database that we used for matching the spectra was filtered so that only one protein per peptide was kept, to control for any difference in protein inference strategies used by our compared protein summarization methods. There is currently no consensus on how to handle multiple proteoforms in bottom-up proteomics. Hence, we believe that protein inference strategies that can account for multiple proteoforms would greatly benefit the field by improving the quality of the quantitative analysis.

We also see some differences in how DIA and DDA peptide-level abundance data appear. For instance, there are more missing values in the DDA data than in the DIA data. We addressed this issue by providing an alternative method for estimating the censoring function for missing values in Triqler.

It is quite hard to evaluate how the performance of a data processing pipeline is influenced by its components. This should not stop the field from trying to establish the features of the different processing steps.^6,29,30^ Unbiased comparisons of software tools are challenging for several reasons.^29^ Methods can be assessed by scientists lacking relevant expertise; the tested methods may be lacking sufficient documentation, and the interpretation of test results may be subjective.^31−34^ By using the same data set, we can ensure that the data set is processed consistently and further the analysis by extending it to protein summarization procedures.

Last, we want to highlight the benefit and importance of data sets such as the one provided by Navarro et al.^6^ These benchmarking data sets make it easy for the scientific community to investigate computational tools by providing a golden standard and significantly facilitating benchmark studies.

## Supporting Information Available

Table S1, protein count in the Uniprot FASTA protein database; Table S2, number of identified peptides and proteins for the ID workflow; Table S3, number of identified peptides and proteins for the PS workflow; Figure S1, standard deviation of peptide abundance as a function of mean abundance in DIA experiments; Figure S2, comparison of actual missing values against fit to the censoring distribution used in Triqler; Figure S3, comparison of the reported log2-fold change between Triqler and the compared methods; Figure S4, the compared methods’ ability to distinguish differentially abundant proteins when applying protein-level fold-change thresholds; Figure S5, the compared methods’ ability to distinguish differentially abundant proteins when not applying fold change treshholds; Figure S6, the compared methods’ ability to distinguish differentially abundant proteins when removing shared peptides after database matching; Figure S7, comparison of reported fold change distributions; and Figure S8, comparison of calibration of the compared summarization methods (PDF)
