# Supplementary material for: Triqler for Protein Summarization of Data from Data-Independent Acquisition Mass Spectrometry
Source: J Proteome Res. 2023 Mar 29;22(4):1359–66. doi: 10.1021/acs.jproteome.2c00607 (PMC10088044; doi:10.1021/acs.jproteome.2c00607)
Supplement: Supplementary file 1 — pr2c00607_si_001.pdf [file pr2c00607_si_001.pdf]

Supplement to: “Triqler for Protein Summarization of Data from Data Independent Acquisition Mass Spectrometry”

Patrick Truong[1], Matthew The[2], and Lukas Käll[1,*]

[1]Science for Life Laboratory, School of Engineering Sciences in Chemistry, Biotechnology and Health, Royal Institute of Technology – KTH, Solna, Sweden
[2]Chair of Proteomics and Bioanalytics, Technical University of Munich (TUM), Freising, Germany
[*]Corresponding author, lukas.kall@scilifelab.se

February 23, 2023

Note S1: Supplementary figures and tables

	Unfiltered	no_shared
All	31 055	30 456
E. Coli	4 391	4 306
Human	20 614	20 302
Yeast	6 050	5 848

Table S1: **Protein count in the Uniprot FASTA protein database.** The database is a FASTA file with one protein sequence per gene for each species (UP000005640, UP000000625, and UP000002311. Acquired on 2021-06-16). We further filtered the sequences to assure that no two proteins shared tryptic peptides longer than 7 amino acids. The number of sequences remaining after this operation is reported in the no_shared column.

ID workflow						
Condition	A			B		
Filename	002-Pedro	004-Pedro	006-Pedro	003-Pedro	005-Pedro	007-Pedro
Peptides	12 934	13 819	13 063	12 023	14 858	15 208
Proteins	2 252	2 321	2 243	2 159	2 427	2 433

Table S2: **Number of identified peptides and proteins for the ID workflow.** A peptide-level FDR at 0.01 was obtained using a an *m_score* cutoff at 0.00079 computed by setting the desired peptide-level FDR with `mscore4pepfdr` in the `SWATH2stats` package.

PS workflow						
Condition	A			B		
Filename	002-Pedro	004-Pedro	006-Pedro	003-Pedro	005-Pedro	007-Pedro
Peptides	20 880	20 653	20 907	21 118	21 192	21 137
Proteins	3 228	3 224	3 240	3 243	3 255	3 249

Table S3: **Number of identified peptides and proteins for the PS workflow** A peptide-level FDR at 0.01 was used for the purpose of reporting these figures.

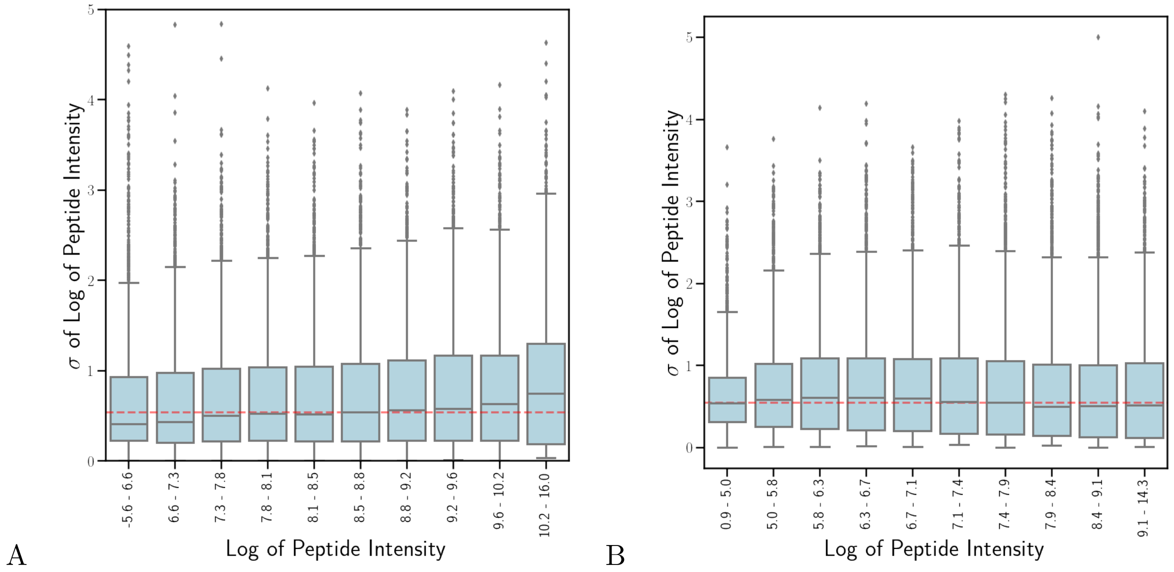

Figure S1: **Standard deviation of peptide abundance as a function of mean abundance in DIA experiments.** We performed quantile binning with 10 bins using pandas qcut function and plotted the standard deviation across samples as a function of the mean of every peptide intensity in the TripleTOF6600 section of the LFQ Bench set. The bins are constructed so that each bin contains an equal amount of data points and the unequal bin sizes are an effect of the quantile binning procedure. The x-axis value shows the bin ranges. We used a log-log scale for abundances derived by the (A) ID pipeline and (B) PS pipeline. (A) show a slight increase in peptide intensity standard deviation as we increase the mean peptide intensity. In (B) we observe a nearly uniform offset in standard deviation across the intensity scale, demonstrating that (B) holds the Triqler assumption $\log(\sigma) \approx \log(\mu) + \log(k)$ and hence $\sigma \approx \mu k$ very well. While (A) does not hold the assumption as well, Triqler is still used to analyse this data.

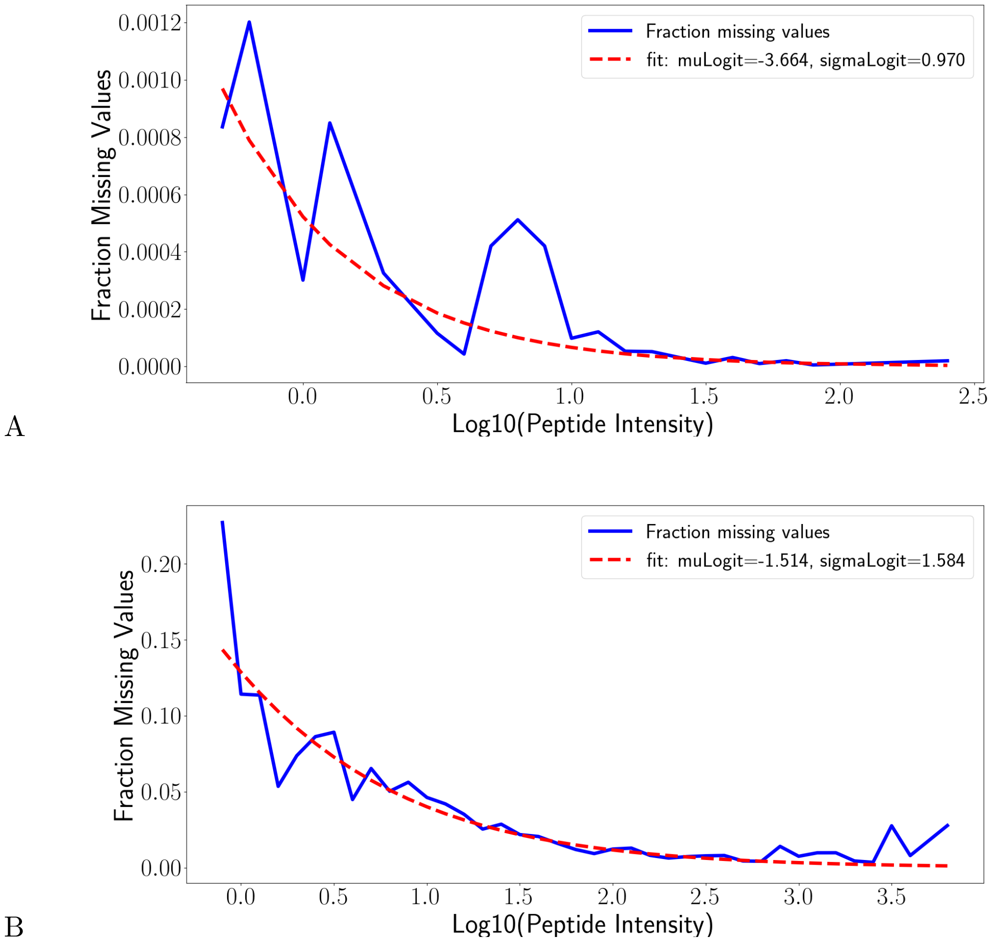

Figure S2: **Comparison of actual missing values against fit to the censoring distribution used in Triqler.** We modified Triqler to use an alternative censoring function for DIA data. To compute the parameters of the function, we imputed the missing values as the mean of sample peptide intensities and used these imputed values to approximate the missingness for a given intensity. We binned the intensities to and plotted the fraction of missing values for each intensity range. The curve_fit function from scipy.optimize was used to fit the values against the censoring function using the variance of a binomial distribution to weight for the uncertainty for each bin. The observed and fitted fractions of missing values are plotted for (A) ID pipeline and (B) PS pipeline.

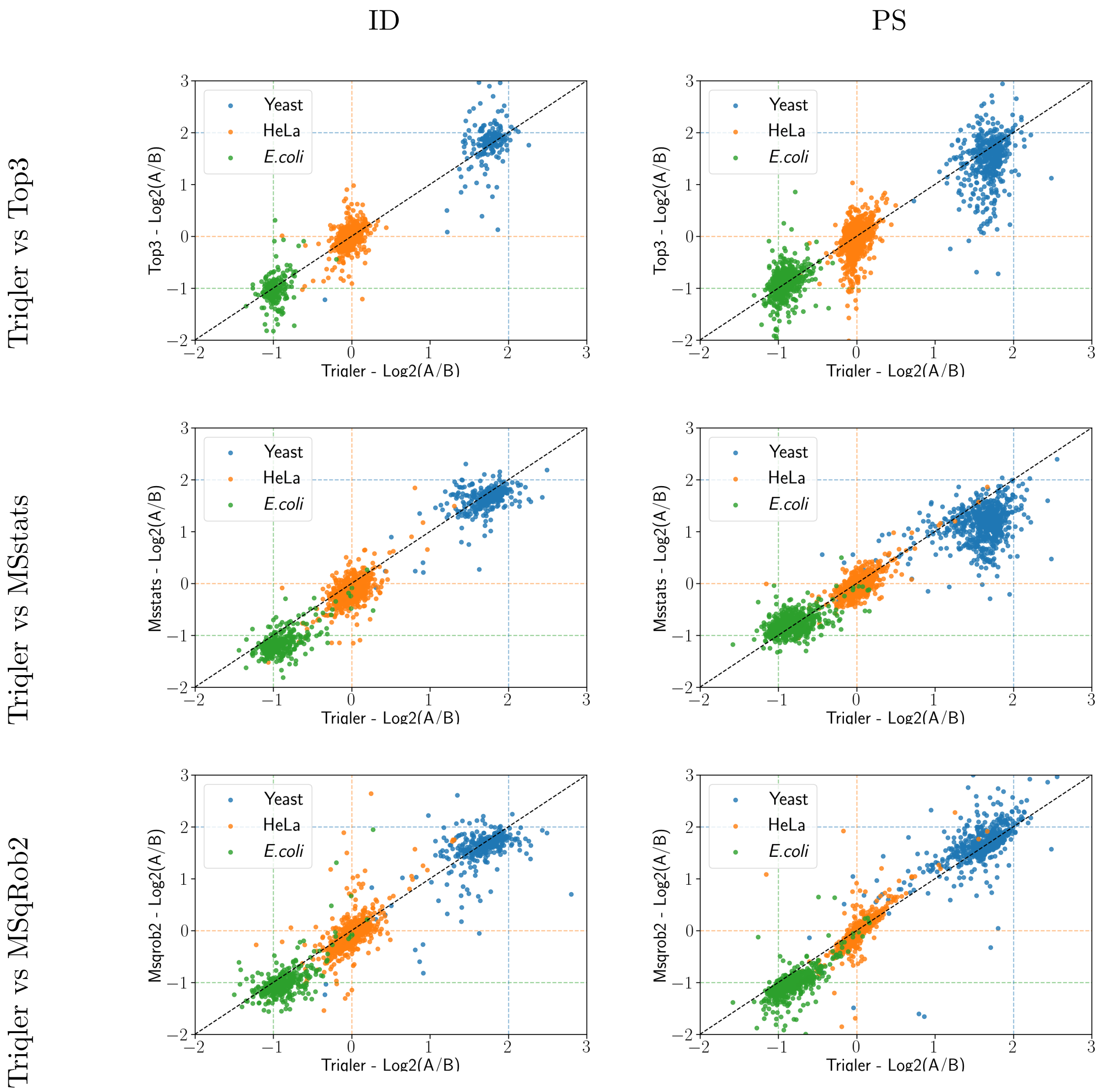

Figure S3: **Comparison of reported log2-fold change between Triqler and the compared methods.** Top3 proteins have a higher variance in log2-fold changes than Triqler. MSstats and MSqRob2 has a bias for the *E. coli* group.

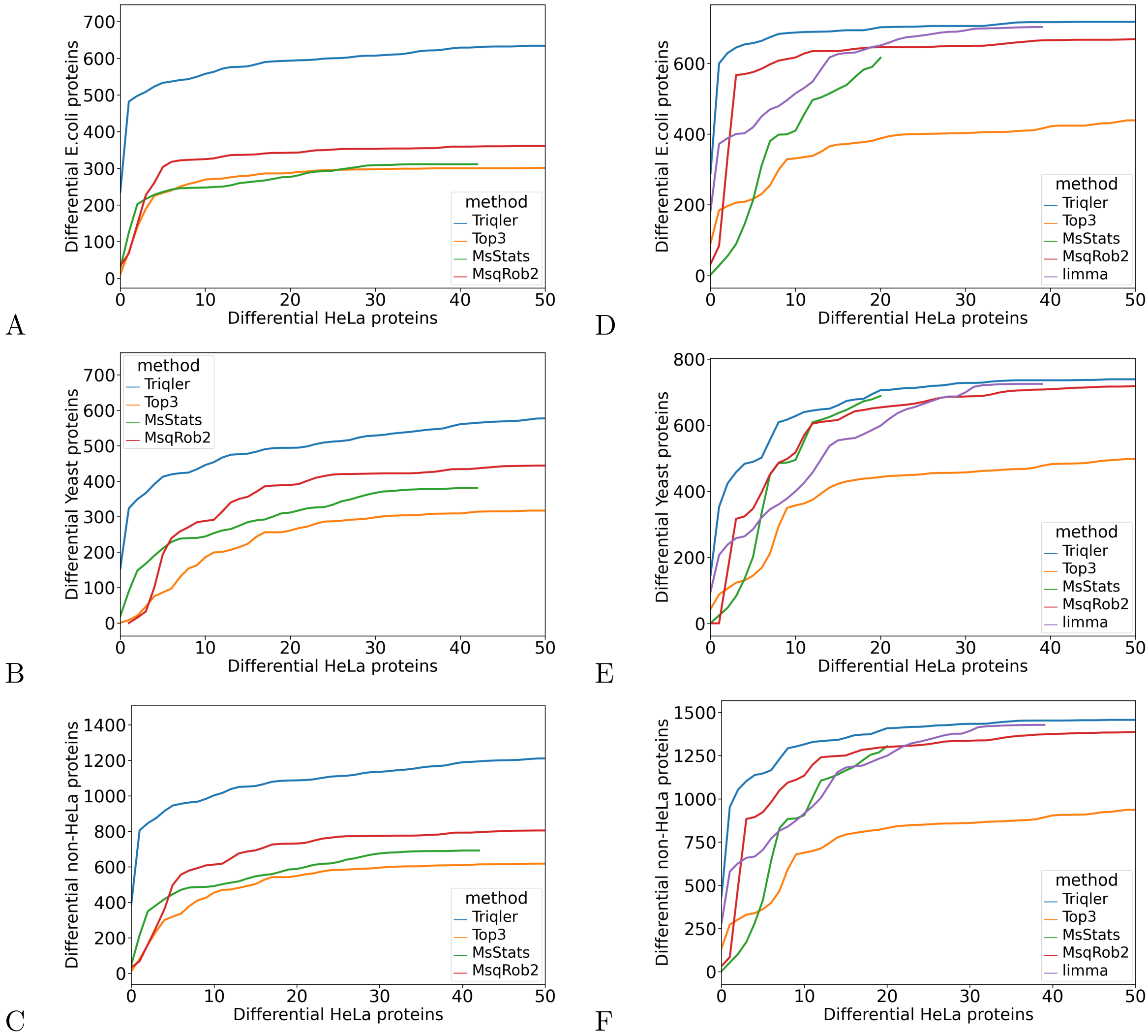

Figure S4: **The compared methods' ability to distinguish differentially abundant proteins when applying protein-level fold-change tresholds.** We plotted the number of reported differentially abundant (A,D) *E. Coli*, (B,E) Yeast proteins and (C,F) the sum of them as a function of the number of proteins from the HeLa background when sorting according to significance for (A-C) ID pipeline and (D-F) PS pipeline. For the test, we selected a fold-change evaluation of 0.51 for Triqler and fold-change threshold of 0.51 for Top3, MSstats, MSqRob2 and limma.

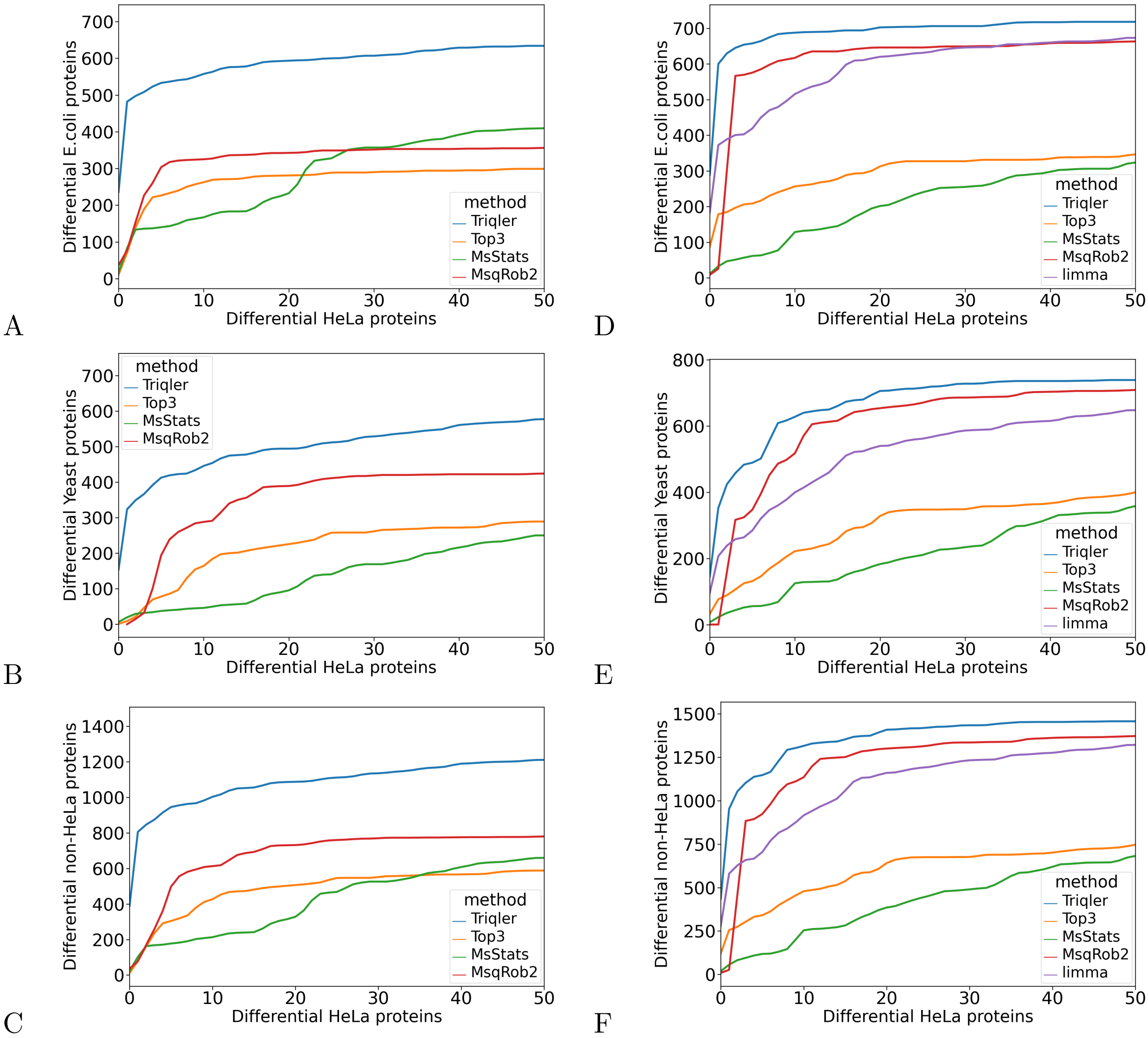

Figure S5: **The compared methods' ability to distinguish differentially abundant proteins when not applying fold change treshholds.** We plotted the number of reported differentially abundant (A,D) *E. Coli*, (B,E) Yeast proteins and (C,F) the sum of them as a function of the number of proteins from the HeLa background when sorting according to significance for (A-C) ID pipeline and (D-F) PS pipeline. For the test, we selected a fold-change evaluation of 0.51 for Triqler, but did not apply any fold-change threshold for Top3, MSstats, MSqRob2, and limma.

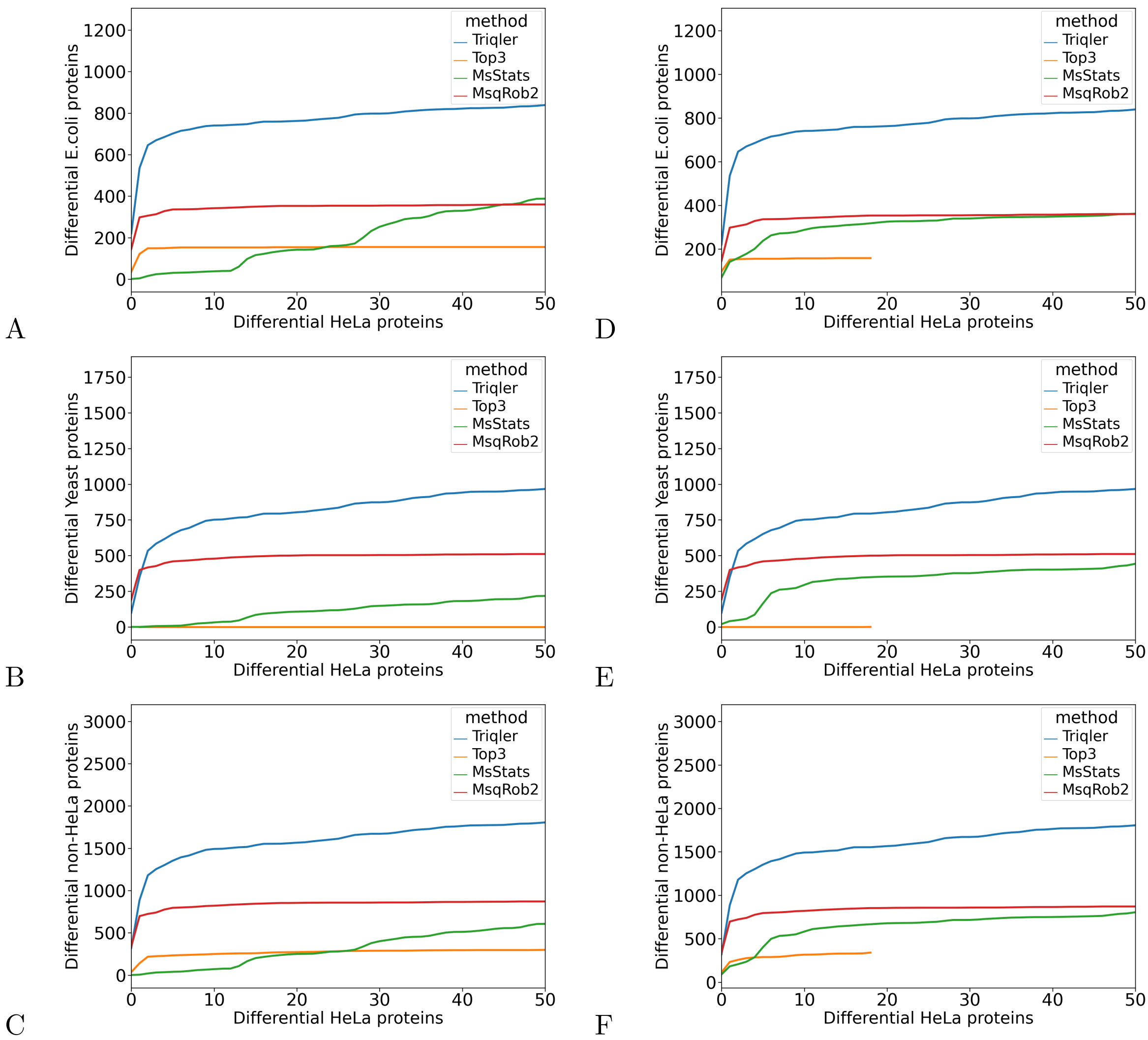

Figure S6: **The compared methods' ability to distinguish differentially abundant proteins when removing shared peptides after database matching.** In the previously described work we removed shared peptides pior to database searching. To validate that this do not cause problems with the search procedure themselves, we here searched against full databases and removed shared peptides from the result files after database matching. As before, the number of reported differentially abundant *E. Coli* and Yeast proteins as a function of the number of proteins from the HeLa background when sorting according to significance for (A-C) without fold-change threshold and (D-F) with fold-change threshold for ID pipeline. For the test, we selected a fold-change evaluation of 0.51 for Triqler and fold-change threshold of 0.51 for Top3, MSstats, and MSqRob2.

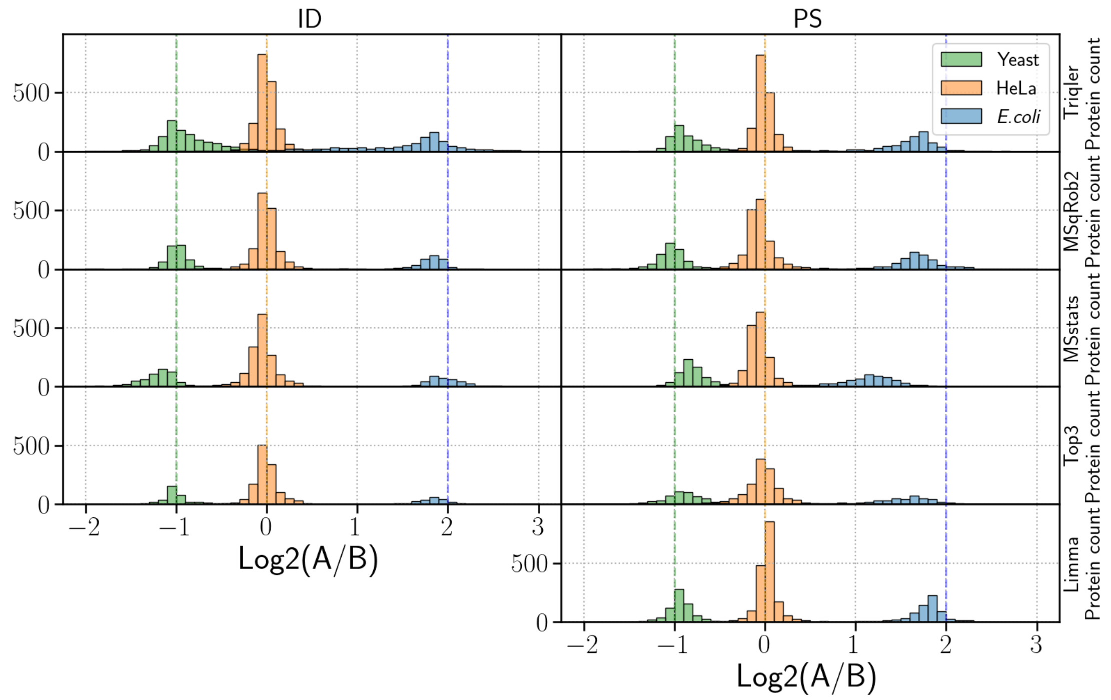

Figure S7: **Comparison of reported fold change distributions.** We can see that the Triqler and Top3 log2(A/B) empirical distributions have apexes that are more centered toward the true lysate values, which are indicated by the dashed lines. The apexes for Triqler have higher protein count than MSqRob2, MSstats and Top3. This shows that there are more proteins closer to the true values identified by Triqler than the other methods.

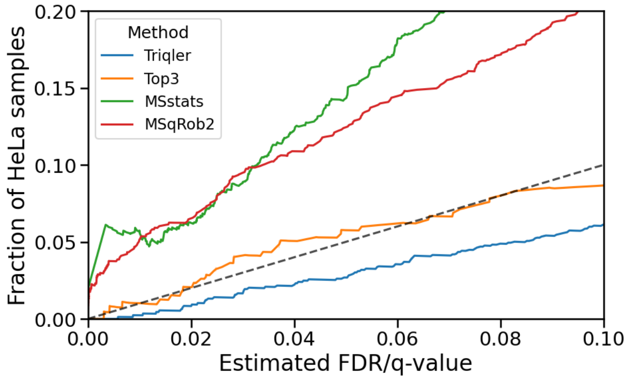

A

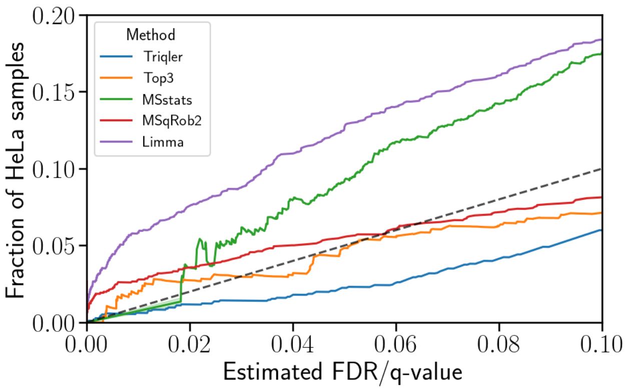

B

Figure S8: **Comparison of calibration of the compared summarization methods.** We plotted the fraction of reported differentially abundant HeLa proteins as a function of q value for (A) ID pipeline and (B) PS pipeline.
